# Current and projected incidence trends of pediatric-onset inflammatory bowel disease in Germany based on the Saxon Pediatric IBD Registry 2000–2014 –a 15-year evaluation of trends

**DOI:** 10.1371/journal.pone.0274117

**Published:** 2022-09-09

**Authors:** Ivana Kern, Olaf Schoffer, Thomas Richter, Wieland Kiess, Gunter Flemming, Ulf Winkler, Jürgen Quietzsch, Olaf Wenzel, Marlen Zurek, Ulf Manuwald, Janice Hegewald, Shi Li, Jens Weidner, Jan de Laffolie, Klaus-Peter Zimmer, Joachim Kugler, Martin W. Laass, Ulrike Rothe

**Affiliations:** 1 Department of Health Sciences/Public Health, Institute and Policlinic for Occupational and Social Medicine, Faculty of Medicine „Carl Gustav Carus”, TU Dresden, Dresden, Germany; 2 Center for Evidence-based Healthcare, University Hospital and Faculty of Medicine “Carl Gustav Carus”, TU Dresden, Dresden, Germany; 3 Clinic for Children and Adolescents, Hospital St. Georg, Leipzig, Germany; 4 Center for Pediatric Research, Department of Women and Child Health, Hospital for Children and Adolescents, University of Leipzig, Leipzig, Germany; 5 Clinic for Children and Adolescents, Hospital Bautzen, Oberlausitz-Hospitals, Bautzen, Germany; 6 Clinic for Children and Adolescents, DRK Hospital Lichtenstein, Lichtenstein, Germany; 7 Clinic for Children and Adolescents, Helios Hospital Aue, Bad Schlema, Germany; 8 Department of Occupational, Social and Environmental Epidemiology, Institute and Policlinic for Occupational and Social Medicine, Faculty of Medicine „Carl Gustav Carus”, TU Dresden, Dresden, Germany; 9 Center for Medical Informatics, Institute for Medical Informatics and Biometry, Faculty of Medicine “Carl Gustav Carus”, TU Dresden, Dresden, Germany; 10 Department of General Pediatrics, Children’s Gastroenterology/ Hepatology/ Nutrition, Justus-Liebig-University Gießen, CEDATA-GPGE Working Group, Gießen, Germany; 11 University Hospital for Children and Adolescents, Faculty of Medicine “Carl Gustav Carus”, TU Dresden, Dresden, Germany; University of Lille, FRANCE

## Abstract

**Aims:**

An increasing number of children and adolescents worldwide suffer from inflammatory bowel disease (IBD) such as Crohn’s disease (CD) and ulcerative colitis (UC). The present work aims to investigate the incidence, prevalence and future trends of IBD in children and adolescents in Saxony, Germany.

**Methods:**

The Saxon Pediatric IBD Registry collected data on patients up to 15 years of age from all 31 pediatric hospitals and pediatric gastroenterologists in Saxony over a 15-year period (2000–2014). In 2019, an independent survey estimated a registry completeness of 95.7%. Age-standardized incidence rates (ASR) per 100,000 person-years (PY) and prevalence per 100,000 children and adolescents were calculated. Evaluation was also been performed in sex and age subgroups. Joinpoint and Poisson regression were used for trend analyses and projections.

**Results:**

532 patients with confirmed IBD during 2000–2014 were included in the epidemiological evaluation. 63.5% (n = 338) patients had CD, 33.1% (n = 176) had UC and 3.4% (n = 18) had unclassified IBD (IBD-U). The 15-year IBD prevalence was 111.8 [95%-CI: 102.3–121.3] per 100,000. The incidence ASR of IBD per 100,000 PY over the whole observation period was 7.5 [6.9–8.1]. ASR for the subtypes were 4.8 [4.3–5.3] for CD, 2.5 [2.1–2.9] for UC and 0.3 [0.1–0.4] for IBD-U. The trend analysis of ASR using the joinpoint regression confirmed a significant increase for incidence of IBD as well as CD. For IBD, the ASR per 100,000 PY increased from 4.6 [2.8–6.3] in 2000 to 8.2 [7.5–13.6] in 2014; projected incidence rates for IBD in Germany are 12.9 [6.5–25.5] in the year 2025 and 14.9 [6.7–32.8] in 2030, respectively. Thus, the number of new IBD diagnoses in Germany would more than triple (325%) in 2030 compared to 2000. The increase is expected to be faster in CD than UC, and be more in males than in females. The expected number of newly diagnosed children with IBD in Germany is projected to rise to about 1,584 [1,512–1,655] in 2025, and to about 1,918 [1,807–2,29] in 2030.

**Conclusion:**

The incidence of IBD in children and adolescents in Saxony increased at a similar rate as in other developed countries during the observation period. Given this trend, the health care system must provide adequate resources for the care of these young patients in the future.

## Introduction

An increasing number of people worldwide, including children, are suffering from inflammatory bowel disease (IBD) [1–3]. In the 21st century, the major increase is concentrated in newly industrialized countries [4–7].

IBD (ICD-10-GM/2013: K50-K52) [8] is grouped into Crohn’s disease (CD) [9], ulcerative colitis (UC) [10], and unclassified IBD (IBD-U). A common feature of IBD is the continuously occurring or relapsing inflammatory changes of the digestive tract. A complete and permanent cure is currently not possible.

IBD is an immune-mediated disease whose causes were mostly seen in lifestyle (e.g. stress, smoking), industrialization of nutrition, and environmental influences [11–15]. Also, in the first decade of this century, genetic researchers published meta-analyses showing dozens of genes and gene loci associated with IBD [16, 17]. Mutations/polymorphisms of these genes appear to be related to the disruption of epithelial barrier function and inflammatory processes of intestinal bacterial flora [18–20].

The younger the children are when IBD is diagnosed, the greater the importance of genetics for the course and severity of the disease. This is especially true for very early-onset IBD (VEO-IBD), where the diagnosis is made before the age of six [21].

Epidemiological data describe a geographic north-south gradient of IBD disease incidence [22, 23], similar to that observed in other autoimmune diseases, e.g., type-1-diabetes [24]. The prevalence is the highest in northern Europe, North America, and in the southern hemisphere, Australia [1–5, 25]. In about 25–30% of all IBD patients disease onset occurs before the age of 18 [1, 26]. Pediatric IBD incidence trend mostly increases or stagnates [1, 2], some studies describe decrease of age at onset [27, 28]. The incidence of pediatric IBD per 100,000 varies greatly by geographical region, with reported incidences ranging from 0.5 to 23 for IBD, 0.1 to 13.9 for CD, 0.3 to 15 for UC, and 0.0 to 3.6 for IBD-U [2]. Germany, which is located in Western Europe, belongs geographically to regions with the highest IBD burden.

Prior to 2000, clear epidemiological data on the incidence of pediatric IBD in Germany over a longer period was lacking. Consequently, the Saxon Pediatric IBD Registry was established in 2000 to gather reliable and valid epidemiological population-based data. This registry is the only population-based pediatric database in Germany, and aims to monitor and answer IBD-related epidemiological (e.g., trends) and clinical (e.g., disease location) questions based on a complete capture of all patients in Saxony [29, 30].

## Aims

The aim of this article was to present the population-based epidemiological pediatric IBD data for a defined German region (German federal state of Saxony) over a period of 15 years. We aimed to show incidence and prevalence trends of IBD in childhood and adolescence and the distribution patterns of CD and UC by age and sex. We projected the probable incidence trends and number of patients with CD and UC in sex and age groups in Germany using statistical models of our registry data.

Our research results will contribute to quality of care, healthcare planning, and the development of pediatric guidelines. These guidelines should help to standardize the diagnosis and therapy of pediatric IBD, to improve the quality of life of patients and their families, and serve as a basis for further education and public relations.

## Methods

### Saxon Pediatric IBD Registry

The Saxon Pediatric IBD Registry collected population-based data prospectively during the 15-year period between 2000 and 2014. The defined territory was the German federal state of Saxony (18,415 km^2^, 4.4 to 4.1 Mio inhabitants). Between 550,835 (in 2000) and 436,305 (in 2005) children under 15 years lived in this area during the observation period. Annual population data were obtained from the State Statistical Office of Saxony and Federal Statistical Office of Germany [31, 32].

All 31 children’s hospitals in Saxony contributed data to the registry. Consequently, all children and adolescents with IBD who were diagnosed and treated by specialized pediatric gastroenterologists in a children’s hospital or an outpatient clinic in Saxony since 2000 were registered. Treating hospitals sent completed standardized forms to the Faculty of Medicine “Carl Gustav Carus” of the Technische Universität Dresden (TUD). Written informed consent was obtained from the parents. Between 2005 and 2008, the Saxon registry was a part of the German CEDATA. Thanks to this cooperation, the Saxon data was supplemented with information from the German CEDATA database [33]. In 2017–2020, the data were completed, sorted, complexly validated, and evaluated by the Department of Health Sciences/Public Health at the TUD. Only those patients who lived in Saxony (according to the residential postal code) [34] and had a confirmed IBD diagnosis in the 15 years period (January 1, 2000 to December 31, 2014) were considered. In Germany, children and adolescents up to the age of 18 years are usually cared for and treated by pediatricians both on an outpatient and inpatient basis. Nevertheless, in adolescents older than 15 years of age, it is possible that an adult gastroenterologist has cared for them [35]. With the aim of guaranteeing the highest data quality, only age groups up to 15 years were evaluated epidemiologically in our study. Results were examined in total and in two age groups (AG): 0–9 and 10–14 years [36]. Additionally, the younger AG was further differentiated into two smaller AG: 0–4 and 5–9 years. The use of these typical age classes facilitated good comparability with studies from other countries.

The most recent confirmed diagnosis (according to the Porto criteria) [37, 38] and the date of initial diagnosis were validated for calculations. IBD patients were defined as patients who met the endoscopic, histologic, diagnostic imaging, clinical and laboratory parameters according to the Lennard-Jones criteria [39], since 2005 according to the Porto criteria [37, 38]. After the diagnosis of IBD (CD, UC or IBD-U) was confirmed, patient data were transmitted to the registry using the “Initial registration form” (S1 and S2 Appendices). This data form included basic characteristics, family history, and the date of diagnosis. At the initial and every subsequent pediatric gastroenterologist consultation (at least once a year), a “Documentation form” (S3 and S4 Appendices) was sent to the registry. Thus, the follow-up was practiced with this form, and periodic validation and completion of the data was performed. Biannual meetings of the clinicians treating children with IBD were held to establish a unified approach to diagnostic and treatment criteria. At these meetings, the patients’ data and the validation of the registry were discussed.

The questionnaires were further developed and improved over the years. In order to process the data flow efficiently, all documents were transferred into a machine-readable layout; questionnaires were scanned and saved as images. A special fax number was set up on a separate fax server, and specially developed scripts enabled automatic, computer-supported data acquisition.

The data in the database were reviewed annually with the aim to confirm patients’ diagnoses and acquire missing information (hospitals were asked for a detailed patient chart review). In a few cases of ambiguity, a follow-up telephone call to the physician was carried out to verify some data. Patients were excluded from the analysis if a clear IBD diagnosis could not be established. We searched the data for duplicate reports and eliminated these from the registry prior to evaluation.

#### Completeness of the registry

The completeness of the registry was confirmed for the years between 2008 and 2014 using the capture-recapture method (Petersen-Lincoln) [40, 41]. This was accomplished by means of an independent survey (second data source) in a defined region of Saxony. For the final evaluation, responses from 254 medical practices (pediatricians, internists and gastroenterologists) were enrolled in a second data source survey. The completeness of the Saxon Pediatric IBD Registry was ascertained as 95.7% [95% CI: 90.2%-100%] [42]. Earlier second data surveys have also certified the high quality of the registry data. In the previous observation period until 2009 the completeness was determined to be 96.7% [28].

### Statistical analysis

**Fig 1:**
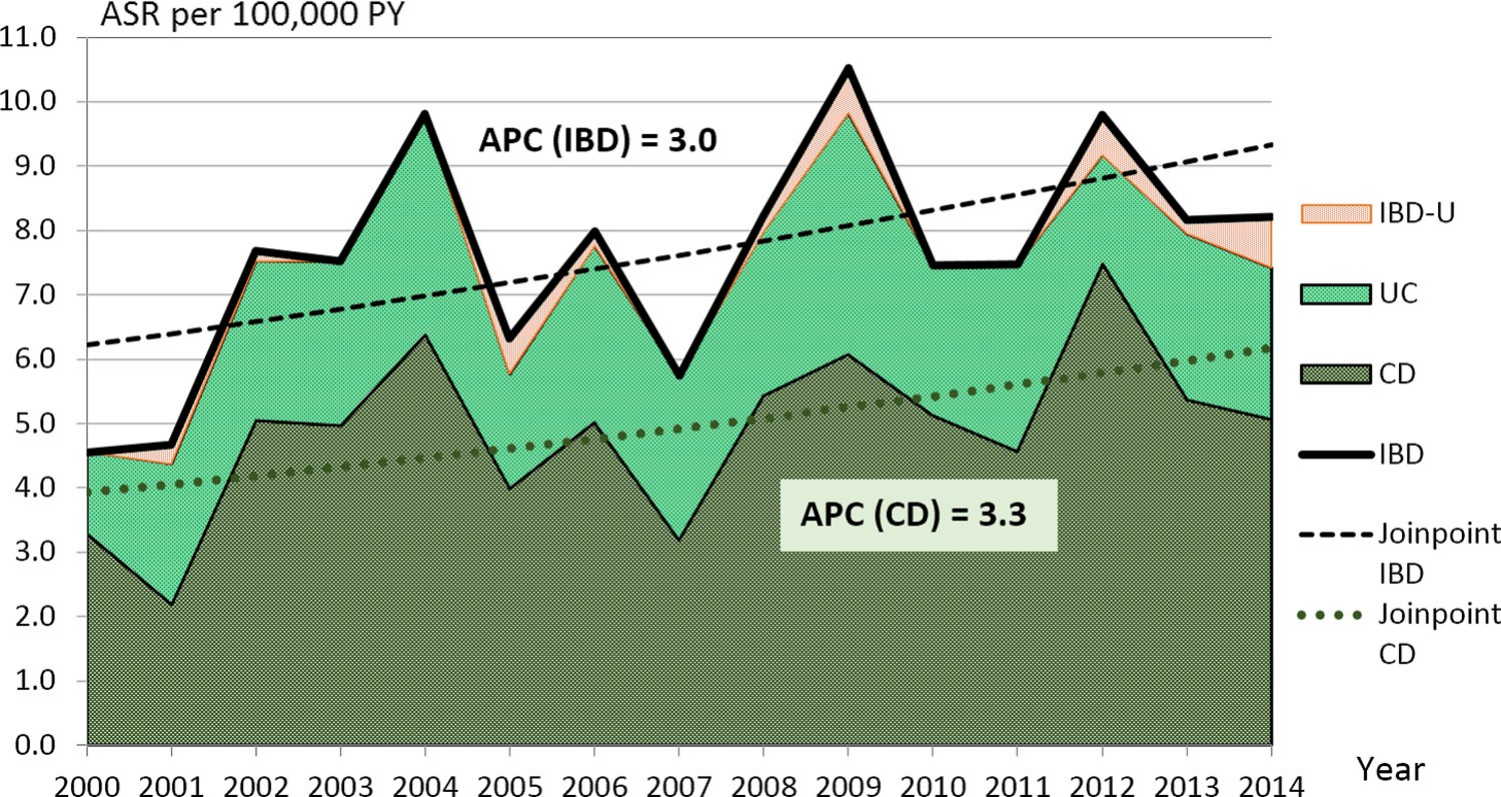
Incidence trends of age-standardized incidence rates (ASR per 100,000 PY) in Saxony, Germany by joinpoint regression, stacked presentation with IBD and CD joinpoint trend lines and corresponding annual percent change (APC) values. Inflammatory bowel disease (IBD), Crohn’s disease (CD), ulcerative colitis (UC) and unclassified IBD (IBD-U).

The New European Standard Population (ESP) (WHO 1990, www.gbe-bund.de) was used for the age-standardization of the incidence and prevalence calculations. As prevalence we estimated a period prevalence over the whole 15 registry years related to the general population at the last time point (year). The age-standardized IBD, CD, UC and IBD-U incidence rates (ASR) were presented per 100,000 person-years (PY) and the age-standardized 15-year prevalence per 100,000 children and adolescents [43]. The 95% confidence intervals [CI] of incidence and prevalence estimates and the 95% credibility intervals [CrI] of projections are presented in brackets and were calculated using the Wald principle [44]. The IBD incidence curve in the graph (Fig 1) is obtained by adding the ASR of each disease CD, UC, and IBD-U. Joinpoint regression method was used to analyze the trends and trend changes over time for the incidence rates within the 15 years period and to test their statistical significance [45]. As joinpoint regression was based on logarithmic models, the slope parameter (coefficient of the year variable) can be expressed as annual percent change (APC).

Extrapolation of the joinpoint model allowed projection of the future development of incidence rates for the years 2025 and 2030. Results in subgroups were compared. The number of children with IBD in 2025 and 2030 was modeled by means of Poisson regression using the federal-state-specific population projection model of the German Statistical Office (variant 1) for Saxony [32] for the corresponding age and sex cohort in corresponding years as an offset. The occurrence of over dispersion was examined.

The duration of the observation period was calculated for each patient and evaluated using median and interquartile range (IQR, given in brackets).

Calculations were carried out with the Joinpoint Regression Program (Version 4.2.0.2, Statistical Research and Applications Branch, National Cancer Institute, Bethesda, Maryland, USA) and with R-Program (Version 3.4.3). The level of significance was defined as α = 0.05.

Other statistical evaluation, tables and graphics for this publication were created in SPSS (Version 25, IBM Corporation, Armonk, New York, USA) or Microsoft Excel (Microsoft Corporation, Redmond, WA).

### Compliance with ethical standards

#### Ethics statement

The Ethics Committee of the University of Leipzig (Reg. No. 033/2000) approved the registry design. All procedures have been performed in accordance with the ethical standards of institutional and national research, the Helsinki Declaration or comparable ethical standards. For all underage patients, written parental consent was obtained for the processing and storage of personal and medical data (data protection vote). All patient data were pseudonymized (a biunique identifier/patient number was used within the whole database) and handled confidentially in the database at the TU Dresden in accordance with data protection regulation of Federal Republic of Germany and Europe.

## Results

After a systematic reduction of duplicities and partial reports, 5,581 reporting forms (532 initial registration forms and 5,049 documentation forms) were considered for a total of 532 children with IBD. The number of reports per patient fluctuated between 2 and 74, an average of 10.5 reports were received per patient (CD: 11.7, UC: 8.8, IBD-U: 3.9). 95 patients (17.9%) had only 2 forms. The average observation time for a registry patient was 2.8 years, mean 2.3 (IQR: 0.1–4.3), max. 13.1 years.

### Included patients

Over the entire 15-year period, 532 pediatric patients with an IBD diagnosis confirmed by Porto criteria [37] were reported to the registry. About 76% of the patients were initially diagnosed in one of the 6 specialized centers for pediatric gastroenterology in large cities. Of these, 338 (63.5%) were diagnosed with CD, 176 (33.1%) with UC, and 18 (3.4%) with an unclassified IBD-U.

Among these 532 IBD patients, 312 (58.6%) were male and 220 (41.4%) were female.

The proportion of male and female patients differed for each form of IBD. While the number of male and female UC patients (91 male, 85 female) was nearly equal (51.7% of male), CD occurred predominantly in male patients (61.5% in CD). There were too few patients with IBD-U (n = 18; 72.2% of male) to reliably consider the distribution.

A total of 153 (28.8%) of all pediatric IBD patients evaluated were 0–9 years old and 379 (71.2%) were 10–14 years old at the age of onset using the age-groups (AG) of the Paris classification. In supplementary subgroups 48 (9.0%) were 0–4 years old and 105 (19.7%) were 5–9 years old at the first diagnosis. We found no considerable differences between CD and UC in the distribution of age groups. The youngest patient was 8 months old, and 2 (0.4%) patients were aged up to 1 year. The oldest registry patient evaluated in this publication was almost 15 years of age at diagnosis.

### Incidence rates and prevalence

Incidence rates for the main and additional age groups (AG) are presented in Table 1. The annual rates for each year 2000–2014 show supplementary material (S5 Appendix). The data were also stratified by sex and in 2 age groups as well as in 2 further supplementary age groups.

**Table 1:** Age-standardized incidence rates (ASR) (sex and age-specific) of inflammatory bowel disease (IBD), Crohn’s disease (CD), ulcerative colitis (UC) and unclassified IBD (IBD-U) per 100,000 person-years (PY) in Saxony. Total rates, sex-specific rates and age-specific incidence rates in age groups (AG) per year.

	mean annual population at risk	IBD			CD			UC			IBD-U		
		n	ASR	[95% CI]	n	ASR	[95% CI]	n	ASR	[95% CI]	n	ASR	[95% CI]
**Sex**													
M-AG 0–9	160,603	87	**3.7**	[2.9–4.5]	53	**2.2**	[1.6–2.9]	29	**1.2**	[0.8–1.7]	5	**0.2**	[0.0–0.4]
M-AG 10–14	84,366	225	**17.8**	[17.0–18.6]	155	**12.3**	[11.6–12.9]	62	**4.9**	[4.5–5.3]	8	**0.6**	[0.5–0.8]
**Male**	244,969	312	**8.6**	[7.6–9.5]	208	**5.7**	[4.9–6.5]	91	**2.5**	[2.0–3.2]	13	**0.4**	[0.2–0.6]
F-AG 0–9	153,101	66	**3.0**	[2.2–3.7]	42	**1.9**	[1.3–2.5]	22	**1.0**	[0.6–1.4]	<5	**0.1**	[0.0–0.2]
F-AG 10–14	80,082	154	**12.8**	[12.1–13.5]	88	**7.3**	[6.8–7.9]	63	**5.2**	[4.8–5.7]	<5	**0.3**	[0.2–0.4]
**Female**	233,183	220	**6.4**	[5.6–7.2]	130	**3.8**	[3.1–4.4]	85	**2.5**	[1.9–3.0]	5	**0.1**	[0.0–0.3]
**AG–all patients**													
AG 0–9	313,704	153	**3.3**	[2.8–3.9]	95	**2.1**	[1.7–2.5]	51	**1.1**	[0.8–1.4]	7	**0.2**	[0.0–0.3]
AG 10–14	164,448	379	**15.4**	[14.8–15.9]	243	**9.9**	[9.4–10.3]	125	**5.1**	[4.8–5.4]	11	**0.5**	[0.4–0.5]
**Total**	**478,152**	**532**	**7.5**	**[6.9–8.1]**	**338**	**4.8**	**[4.3–5.3]**	**176**	**2.5**	**[2.1–2.9]**	**18**	**0.3**	**[0.1–0.4]**
**Supplementary AG**													
M-AG 0–4	84,254	31	**2.5**	[2.1–2.7]	19	**1.5**	[1.3–1.7]	10	**0.8**	[0.6–1.0]	<5	**0.2**	[0.1–0.3]
M-AG 5–9	76,349	56	**4.9**	[4.5–5.3]	34	**3.0**	[2.6–3.3]	19	**1.7**	[1.4–1.9]	<5	**0.3**	[0.2–0.4]
F-AG 0–4	80,249	17	**1.4**	[1.2–1.6]	10	**0.8**	[0.7–1.0]	6	**0.5**	[0.4–0.6]	<5	**0.1**	[0.0–0.1]
F-AG 5–9	72,852	49	**4.5**	[4.1–4.9]	32	**3.0**	[2.6–3.3]	16	**1.5**	[1.2–1.7]	<5	**0.1**	[0.0–0.2]
AG 0–4	164,502	48	**2.0**	[1.8–2.1]	29	**1.2**	[1.0–1.3]	16	**0.7**	[0.6–0.8]	<5	**0.1**	[0.1–0.2]
AG 5–9	149,201	105	**4.7**	[4.4–5.0]	66	**3.0**	[2.7–3.2]	35	**1.6**	[1.4–1.7]	<5	**0.2**	[0.1–0.2]

M = male, F = female; source of population data [31].

As a result of observing children and adolescents up to 15 years of age over the 15-year period, recent 15-year prevalences could be determined (see Table 2).

**Table 2:** Age-standardized 15-year prevalence (Prev.) (sex and age specific) of inflammatory bowel disease (IBD), Crohn’s disease (CD), ulcerative colitis (UC) and unclassified IBD (IBD-U) per 100,000 in Saxony.

Cohort	population at risk over 15 years	IBD		CD		UC		IBD-U	
		Prev.	[95% CI]	Prev.	[95% CI]	Prev.	[95% CI]	Prev.	[95% CI]
**Sex**		** **		** **		** **		** **	
M-AG 0–9	2,409,044	**49.7**	[47.0 − 52.4]	**30.3**	[28.2 − 32.4]	**16.6**	[15.0 − 18.1]	**2.9**	[2.2 − 3.5]
M-AG 10–14	1,265,485	**276.5**	[273.3 − 279.7]	**190.5**	[187.8 − 193.2]	**76.2**	[74.5 − 77.9]	**9.8**	[9.2 − 10.4]
**Male**	3,674,529	**128.1**	[113.9 − 142.3]	**85.7**	[74.0 − 97.3]	**37.2**	[29.5 − 44.8]	**5.3**	[2.4 − 8.1]
F-AG 0–9	2,296,509	**40.0**	[37.5 − 42.5]	**25.5**	[23.5 − 27.5]	**13.3**	[11.9 − 14.7]	**1.2**	[0.8 − 1.6]
F-AG 10–14	1,201,236	**197.9**	[195.1 − 200.7]	**113.1**	[111.0 − 115.2]	**80.9**	[79.2 − 82.7]	**3.9**	[3.5 − 4.2]
**Female**	3,497,745	**94.7**	[82.1 − 107.2]	**55.8**	[46.2 − 65.4]	**36.7**	[28.9 − 44.6]	**2.1**	[0.3 − 4.0]
**AG—all patients**		** **							
AG 0–9	4,705,553	**44.9**	[43.1 − 46.8]	**27.9**	[26.5 − 29.4]	**15.0**	[13.9 − 16.0]	**2.0**	[1.7 − 2.4]
AG 10–14	2,466,721	**238.1**	[235.9 − 240.2]	**152.6**	[150.9 − 154.3]	**78.5**	[77.3 − 79.7]	**6.9**	[6.5 − 7.3]
**Total**	**7,172,274**	**111.8**	**[102.3 − 121.3]**	**71.1**	**[63.5 − 78.7]**	**37.0**	**[31.5 − 42.4]**	**3.7**	**[2.0 − 5.5]**
**Supplementary AG**		** **							
M-AG 0–4	1,263,805	**34.1**	[33.1 − 35.1]	**20.9**	[20.1 − 21.7]	**11.0**	[10.4 − 11.6]	**2.2**	[2.0 − 2.5]
M-AG 5–9	1,145,239	**64.9**	[63.4 − 66.3]	**39.4**	[38.3 − 40.5]	**22.0**	[21.2 − 22.9]	**3.5**	[3.1 − 3.8]
F-AG 0–4	1,203,731	**19.7**	[18.9 − 20.4]	**11.6**	[11.0 − 12.2]	**6.9**	[6.5 − 7.4]	**1.2**	[1.0 − 1.4]
F-AG 5–9	1,092,778	**59.8**	[58.4 − 61.2]	**39.1**	[37.9 − 40.2]	**19.5**	[18.7 − 20.4]	**1.2**	[1.0 − 1.4]
AG 0–4	2,467,536	**27.1**	[26.4 − 27.7]	**16.4**	[15.9 − 16.9]	**9.0**	[8.7 − 9.4]	**1.7**	[1.5 − 1.9]
AG 5–9	2,238,017	**62.4**	[61.4 − 63.4]	**39.2**	[38.4 − 40.0]	**20.8**	[20.2 − 21.4]	**2.4**	[2.2 − 2.6]

M = male, F = female; source of population data [31]. The numbers of cases (n) in Table 2 are identical to Table 1 and therefore omitted here.

The distribution of 15-year prevalence and incidence rates were similar for individual diseases and sex. However, small differences exist here between the prevalence and incidence within age groups.

### Incidence trend analysis

In 2000, at the beginning of the observation period, the ASR of IBD per 100,000 PY was 4.6 [2.8–6.3], while at the end of this observation period in 2014 the ASR of IBD was 8.2 [5.6–10.8] (see Fig 1 and S5 Appendix).

The trend lines, which were placed over the ASR, clearly document the increase both in IBD and in CD separately. This pattern can also be examined in UC, IBD-U, by sex and in age groups. The slope (APC) was positive in all trend subgroups except UC-female, where incidence remained almost unchanged over time.

Significant increases of incidence rates over the 15 years period were ascertained for IBD-all (APC = +3.0 [0.2–5.8]) and in 4 subgroups: IBD-male, CD-all, CD-male and CD-AG 10–14 (see Table 3). For the subgroup IBD-female segment 2002–2014 a decreasing trend (APC = − 0.9) that was slightly less pronounced for the UC-female subgroup (APC = − 0.1); but neither trend was significant. For IBD-female and IBD-AG 10–14 no continuous trends were observed, and APC values were calculated in 2 distinct time segments with respective joinpoints in 2002. For all other subgroups, positive non-significant values of APC were found. As no joinpoints were found for any of these other groups considered, there is no evidence of a change in trends over time. In IBD-U, there were 6 years with zero number of patients (see S5 Appendix), so APC could not be determined.

**Table 3:** Results of the incidence trend analysis of inflammatory bowel disease (IBD), Crohn’s disease (CD), ulcerative colitis (UC) and unclassified IBD (IBD-U) by sex and age groups (AG) using joinpoint regression and annual percent change (APC) over the period 2000–2014.

disease	sex/ AG		segment start—end	APC	[95% CI]	p-value
CD	all		c. p.	**+ 3.3**	[0.0–6.7]	0.049*
CD	male		c. p.	**+ 4.5**	[0.6–8.5]	0.027*
CD	female		c. p.	+ 1.6	[− 3.2–6.7]	0.486
CD	AG 0–9		c. p.	+ 0.8	[− 5.0–6.9]	0.779
CD	AG 10–14		c. p.	**+ 4.1**	[1.2–7.2]	0.009*
UC	all		c. p.	+ 1.4	[− 2.0–4.9]	0.406
UC	male		c. p.	+ 1.8	[− 3.1–6.9]	0.456
UC	female		c. p.	− 0.1	[− 5.2–5.3]	0.970
UC	AG 0–9		c. p.	+ 1.5	[− 9.7–14.0]	0.794
UC	AG 10–14		c. p.	+ 1.0	[− 2.4–4.5]	0.537
IBD-U		no estimation is possible due to zero values				
IBD	all		c. p.	**+ 3.0**	[0.2–5.8]	0.037*
IBD	male		c. p.	**+ 4.0**	[0.8–7.3]	0.018*
IBD	female		2000–2002	+ 47.8	[− 25.9–194.9]	0.237
IBD	female		2002–2014	− 0.9	[− 4.6–2.9]	0.593
IBD	AG 0–9		c. p.	+ 2.1	[− 4.4–9.0]	0.512
IBD	AG 10–14		2000–2002	+ 32.1	[− 8.7–91.1]	0.124
IBD	AG 10–14		2002–2014	+ 1.1	[− 1.1–3.3]	0.283

c. p. = complete period, CI = confidence interval

* = significant

### Projected trends

Based on the evaluated trends of ASR in the 15-year period 2000–2014 in Saxony and with the help of joinpoint model, it is possible to calculate a prospect on the future ASR for the population at risk (children and adolescents <15 years of age). Projected ASR for the example years 2025 and 2030 are shown in Table 4.

**Table 4:** Projected age-standardized incidence rates (ASR) (sex and age specific) of Crohn’s disease (CD) und ulcerative colitis (UC) per 100,000 person-years (PY) in Saxony for the years 2025 and 2030.

Cohort	CD				UC			
	2025		2030		2025		2030	
	ASR	[95% CrI]	ASR	[95% CrI]	ASR	[95% CrI]	ASR	[95% CrI]
**Sex**			** **				** **	
Male	**13.1**	[4.3 − 40.0]	**16.4**	[4.5 − 59.1]	**3.6**	[1.2 − 11.1]	**3.9**	[1.1 − 14.4]
Female	**5.4**	[1.3 − 23.1]	**5.8**	[1.1 − 31.4]	**2.6**	[0.3 − 23.6]	**2.6**	[0.2 − 32.8]
**Age Groups**	** **		** **		** **		** **	
AG 0–9	**2.6**	[0.2 − 29.0]	**2.7**	[0.2 − 43.7]	**1.8**	[0.1 − 36.1]	**1.9**	[0.1 − 61.4]
AG 10–14	**21.1**	[10.1 − 44.1]	**25.8**	[11.1 − 60.4]	**6.4**	[2.0 − 20.7]	**6.7**	[1.7 − 26.1]
**Total**	**8.8**	**[3.6 − 21.4]**	**10.4**	**[3.7 − 28.8]**	**3.2**	**[1.3 − 7.9]**	**3.5**	**[1.2 − 9.6]**

**Fig 2:**
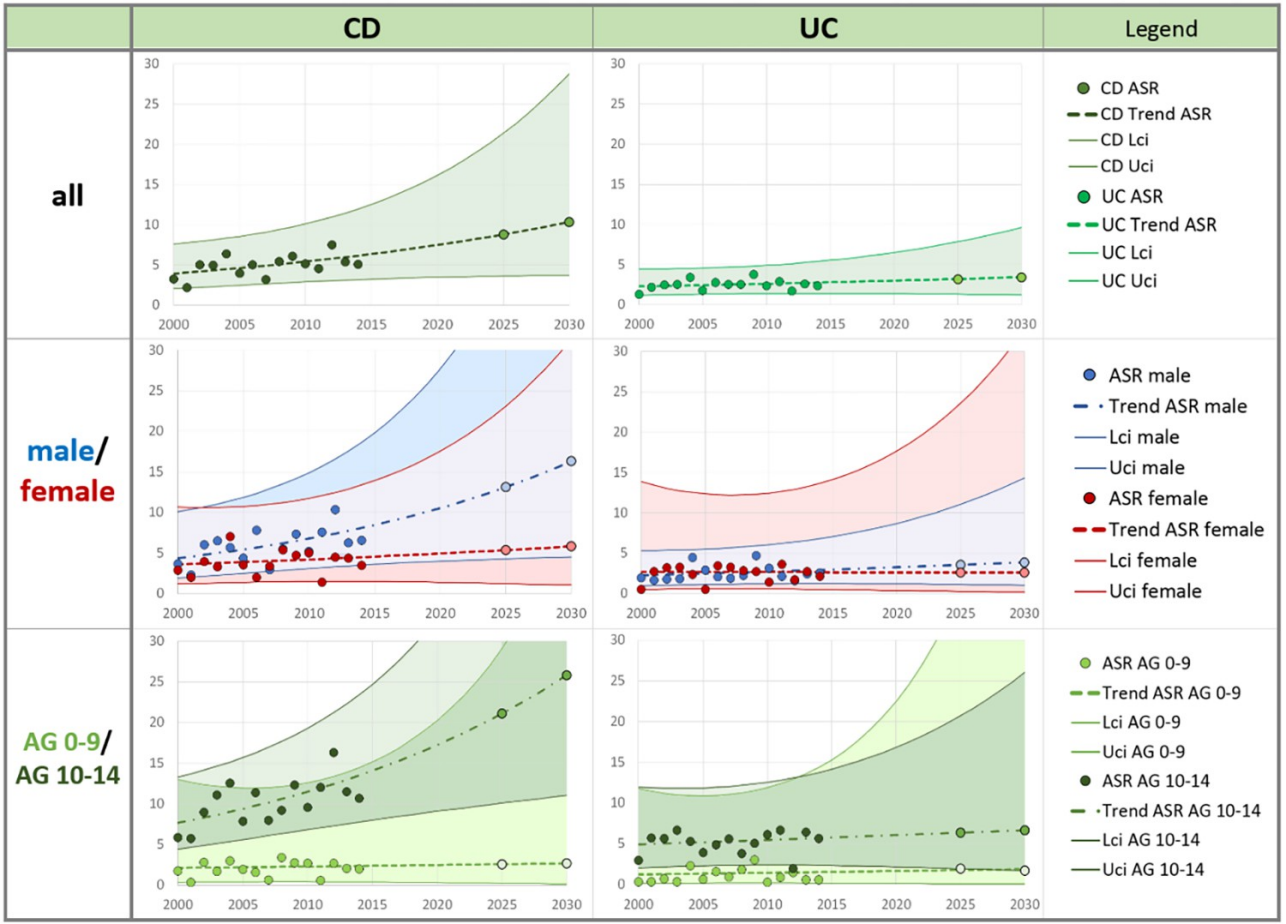
Trends of age-standardized incidence rates (ASR) with projection to 2025, 2030 in Saxony, Germany for Crohn`s disease (CD) and Ulcerative colitis (UC) by sex and age-groups. Points are observed age-standardized incidence rates (ASR), sex-specific rates and age-specific incidence rates in age groups (AG) per 100,000 PY. Dashed line types are incidence trends of ASR by joinpoint regression with projected points (lighter color) in 2025 and 2030. Corresponding 95% credibility intervals [lower limit (Lci)–upper limit (Uci)] are shown.

The expected ASR per 100,000 PY can also be estimated for IBD as 12.9 [6.5–25.5] for 2025 and 14.9 [6.7–32.8] for 2030. IBD-male ASR was determined as 17.7 [7.4–42.0] for the year 2025 and 21.5 [7.9–58.3] for 2030, then for subgroup AG 0–9 as 5.2 [0.7–38.9] for the year 2025 and 5.7 [0.6–58.6] for 2030. The joinpoint regression model calculated trends for the subgroups IBD-female and IBD-AG 10–14 in two segments (see Table 3). As a consequence, the projected IBD rates are certainly higher than the addition of the CD + UC rates of the corresponding subgroup. Incidence trends in individual diseases and subgroups are shown in Fig 2.

Increasing or constant trends are observed in both diseases CD and UC and in all subgroups, with CD showing a much steeper increase in incidence rates than UC. Differences in trends were also noted in separate sex subgroups. A steeper slope was observed among males, although the difference in UC is very small. Much higher incidence rates were found in AG 10–14 than in AG 0–9. Incidence trend is also much steeper in the older AG 10–14 for CD than in all other subgroups.

Narrower credibility intervals for UC-all and UC-male indicate more certainty of the results. The location and steepness of all joinpoint regression curves correspond to the calculated APC values, the 3 steepest increasing trends (CD-all, CD-male and CD-AG 10–14) are significant (see Table 3). For the years 2025 and 2030, expected incidence rates were illustrated on the trend curves, and these rates were presented in Table 4.

Based on the Poisson regression, a projection of the number of children with this disease in Saxony was calculated for the years 2025 and 2030 (Table 5 and S6 Appendix).

**Table 5:** Projected number of children and adolescents <15 years of age at diagnosis with inflammatory bowel disease (IBD), Crohn’s disease (CD) und ulcerative colitis (UC) in Saxony to the years 2025 and 2030 (and by age and sex).

Cohort	Population at risk	Year	IBD		CD		UC	
			n	[95% CrI]	n	[95% CrI]	n	[95% CrI]
**Sex**		** **						
M-AG 0–9	186,000	2025	**13.4**	[12.5 − 14.3]	**9.8**	[9.0 − 10.7]	**2.7**	[2.4 − 3.1]
	175,000	2030	**14.9**	[13.7 − 16.2]	**11.2**	[10.0 − 12.4]	**2.8**	[2.4 − 3.2]
M-AG 10–14	97,000	2025	**30.0**	[27.4 − 32.5]	**21.7**	[19.4 − 24.0]	**6.6**	[5.6 − 7.6]
	97,000	2030	**35.8**	[31.9 − 39.6]	**26.5**	[22.9 − 30.1]	**7.2**	[5.8 − 8.6]
**Male**	283,000	2025	**43.4**	[40.6 − 46.1]	**31.5**	[29.1 − 34.0]	**9.3**	[8.3 − 10.4]
	272,000	2030	**50.7**	[46.6 − 54.8]	**37.7**	[34.0 − 41.5]	**10.0**	[8.6 − 11.5]
F-AG 0–9	177,000	2025	**9.5**	[8.9 − 10.2]	**6.2**	[5.7 − 6.7]	**2.6**	[2.3 − 2.9]
	167,000	2030	**10.6**	[9.7 − 11.5]	**7.0**	[6.3 − 7.8]	**2.6**	[2.2 − 3.0]
F-AG 10–14	92,000	2025	**21.1**	[19.2 − 22.9]	**13.5**	[12.0 − 15.0]	**6.1**	[5.2 − 7.1]
	92,000	2030	**25.2**	[22.4 − 27.9]	**16.5**	[14.3 − 18.8]	**6.8**	[5.5 − 8.1]
**Female**	269,000	2025	**30.6**	[28.6 − 32.5]	**19.7**	[18.1 − 21.3]	**8.7**	[7.7 − 9.7]
	259,000	2030	**35.7**	[32.8 − 38.7]	**23.6**	[21.2 − 26.0]	**9.4**	[8.0 − 10.8]
**AG–all patients**			** **		** **		** **	
AG 0–9	363,000	2025	**23.0**	[21.8 − 24.1]	**16.0**	[15.0 − 17.0]	**5.3**	[4.9 − 5.8]
	342,000	2030	**25.5**	[24.0 − 27.1]	**18.3**	[16.8 − 19.7]	**5.4**	[4.8 − 6.0]
AG 10–14	189,000	2025	**51.0**	[47.8 − 54.2]	**35.2**	[32.4 − 38.0]	**12.7**	[11.4 − 14.1]
	189,000	2030	**60.9**	[56.2 − 65.7]	**43.1**	[38.8 − 47.3]	**14.0**	[12.1 − 15.9]
**Total**	552,000	2025	**74.0**	[70.6 − 77.3]	**51.2**	[48.3 − 54.2]	**18.1**	[16.6 − 19.5]
	531,000	2030	**86.4**	[81.4 − 91.4]	**61.3**	[56.8 − 65.8]	**19.4**	[17.4 − 21.4]

M = male, F = female; source of population data [32].

Patient numbers for CD are expected to increase in total and in all subgroups. Patient numbers for UC are increasing to a lesser extent in total and in the older children (AG 10–14). In the age group A1a (AG 0–9) of the Paris Classification, patient numbers for UC are constant or minimally increasing. The expected number of patients with IBD, which includes CD, UC, and IBD-U, is increasing or constant (one case) in all age, sex, and disease subgroups.

## Discussion

The Saxon Pediatric IBD Registry in Germany (83.4 million inhabitants in 2020) [32] has collected population-based data for the entire federal state Saxony (4.1 million inhabitants in 2020) [31]. The population size of Saxony is comparable to countries like Croatia, Panama, or the US State of Oregon. After the reunification of Germany in 1990, the population of Saxony quickly adopted the so-called western lifestyle, environmental and living conditions, as well as medical and socio-economic factors changed. And during that time the number of IBD cases increased in many pediatric centers in Saxony [28, 30]. An increasing incidence was suspected, but reliable, long-term, and population-based IBD data for children and adolescents in Germany were not available. So, the Saxon Pediatric IBD Registry was established.

In the first published epidemiological evaluation of the registry covering the years 2000–2009, the increasing incidence of IBD was demonstrated [28]. The significant upward incidence trend was confirmed again for the years up to 2014. The total pediatric (age <15) incidence rate of IBD was already 0.3 per 100,000 PY higher than in the first period. Sex differences were also found, which were not yet evident in the first 10 years.

During the 15-year period, the ASR of IBD per 100,000 PY increased significantly from 4.6 [2.8–6.3] in 2000 to 8.2 [5.6–10.8] in 2014. Whether and to what extent the increase of pediatric IBD in Saxony may be due to the modified living conditions, changes to stress levels in everyday life, altered nutrition and other environmental factors, or a result of increased awareness and advanced diagnostic methods after the German reunification is not known and should be considered in further studies.

In general, we observed continuously increasing incidence trends in the overall evaluation of IBD and mostly (but not always) also in the subgroups of CD and UC and separated by sex. IBD in Germany follows the international trend of increasing IBD cases, although different study designs, methods data acquisition, and data sources makes comparisons difficult. Although the incidences of pediatric IBD vary between countries, systematic reviews [1, 2] show a similar general upward trend in recent decades. Similar to our observations in Saxony, these reviews also show differences between CD and UC: an increase in incidence was reported by 60–67% of all studies for CD, but only for 20–46% of the UC studies. This trend has been observed worldwide, including newly industrialized countries and in both adults and children [3, 46–55]. However, the incidence in Western countries has recently begun to stabilize [4, 5].

According to a review by Sýkora et al. 2018 [2], the global incidence rate of pediatric (age<15) IBD per 100,000 PY in 2000–2014 ranged from approximately 0.2 in Latin America (Argentina) to 18.3 in Europa (Spain) and in Canada (age<16; 15.2). For ages <19 years, the world highest incidence rate of IBD was in Europe (Finland: 23.0). The IBD incidence rate in Saxony (7.5 [6.9–8.1]) was in the upper average compared to the rest of the world, but far from above listed extremes of Europe or North America. Internationally, higher incidence rates are usually reported for CD compared to UC. This is also applicable to Saxony. In Europe, a higher incidence was reported in Western European countries, but the incidence is presently increasing quickly in Eastern European countries [6]. A similar trend has been described for other autoimmune diseases, e.g., type-1-diabetes [24, 56, 57].

Comparison of results for CD and UC within Germany is only possible to a very limited extent, since clear epidemiological pediatric data in Germany are rare. The ASR per 100,000 PY from the population based registry in Saxony (CD: 4.8 [4.3–5.3] and UC: 2.5 [2.1–2.9]) were higher than those reported by Ott et al. 2008 (IBD: 4.0, CD: 2.4; UC: 1.1) for a rural region of southern Germany (Oberpfalz in Bavaria) in 2004–2006 [58]. Wittig et al. (2019) recently studied health administrative data and found incidence values among the highest in the literature (in the year 2012 for age<18; IBD: 17.0, CD: 10.3, UC: 6.0) [59] However, due to its procurement design and inclusion of ages <18 years it might not be comparable to the registry in Saxony.

In up to 10–15% of the IBD cases, a clear diagnosis of CD or UC could not be made. These patients were classified with IBD-U. The younger the patient, the more likely IBD-U is diagnosed [60]. In Saxony, only 3.4% of IBD patients were classified with IBD-U. Correspondingly, there was a relatively low incidence rate of 0.3 [0.1–0.4] per 100,000 PY; in the first 10 years of the registry it was 0.2 [0.1–0.3]. This might be related to the long observation time and clarification of the final diagnosis over time. The diagnoses with which the patients were listed in our registry are not the initial diagnoses but the final ones. Correspondingly, the highest number of IBD-U diagnoses was observed in the last year of the registry. Studies in the USA and Western Europe [2] have determined incidence rates of IBD-U for children up to 15 years ranging 0.1–1.2 per 100,000 PY, while many other studies [1, 3–5] do not provide information on the IBD-U.

The proportion of CD to UC was nearly 2:1, and was similar to other pediatric observations, where almost two-thirds of all IBD patients have CD [28, 33, 61]. The sex distribution followed commonly observed patterns. The male to female ratio was about 1:1 for UC, and about 3:2 for CD. In IBD-U it was about 7:3 in favor of males, but this ratio is probably biased by the very small number of IBD-U patients (n = 18). In the literature, this sex ratio is more 1:1 balanced [33]. Unlike pediatric IBD, the number of women with IBD exceeds the number of men among adults older than about 25 years [5].

Distribution of patients by age confirmed results from previous registry publications [28, 62]. 9% of the children younger than 15 years of age registered in the Saxon Pediatric IBD Registry were younger than 5 years, almost 29% were younger than 10 years. It is difficult to compare percentages, because the proportion of young children depends on the age up to which patients are included in the study. Buderus et al. 2015 [33] who analyzed patients up to 18 years old found 23% were age 0–10 years. The incidence is high in older adolescents, higher compared to children and younger adolescents (age <15) [35]. Concerning the onset of disease symptoms, the literature consistently finds 15–25% (up to 30%) of all IBD patients develop the disease before their 18^th^ birthday [63–65].

To provide a complete overview, the age-standardized 15-year prevalence was also determined. The prevalence of IBD per 100,000 children and adolescents up to 15 years in Saxony was 111.8 [102.3–121.3] (CD: 71.1; UC: 37.0) in the years 2000–2014. Comparison with other studies is difficult because complete pediatric data are rare and methodological aspects differ. The highest reported prevalence for IBD per 100,000 persons in the general population are reported in Europe (CD: 322¸ UC: 505) and North America (CD: 319; UC: 249) [3], and another source indicates CD: 213, UC: 294 in Europe [66]. The pediatric (age 2–17) prevalence of IBD from large national databases in the United States (US) was 77.0 per 100,000 in 2016, where CD was twice as prevalent as UC (45.9 vs. 21.6), and the prevalence was higher in males than females [67]. This prevalence values from the US reported by of Ye et al. 2020 were only one-third lower than 15-year prevalence in Saxony. However, prevalence results for adults in Ye et al. 2020 are also about one-third lower than previous results from North America (Molodecky et al. 2011) [3].

Using joinpoint regression we extrapolated incidence rates from the 15-year observation period to project incidence rates until 2030 (Table 4). We found CD incidence is increasing faster than UC, and the projected incidence of CD in males and children aged 10–14 years is increasing more steeply than all other subgroups.

Using population prediction models for the age group up to 15 years from the Statistical Office of Germany and Saxony, we can estimate the future number of pediatric IBD patients in Germany. Germany is expected to have 83.7 million inhabitants in 2025, and a decline to about 83.1 million inhabitants by 2030 is anticipated, of which about 11.8 million will be children younger than 15 years [32]. Based on our trend calculations, and assuming an equal distribution of IBD throughout Germany, we project about 1,600 new pediatric IBD cases will be diagnosed in 2025, and about 1,900 in the year 2030. This is more than twice as many annual new cases than in 2000–2014. So, in the 6-year period 2025–2030, we expect about 10,500 new pediatric IBD patients in Germany who will need adequate care.

An additional trend estimate and a resulting projection of prevalence would be an asset for future analyses, but will only be possible when a longer observation period is available.

The majority of patients are diagnosed early in life, many in childhood, and the incidence continues to rise; therefore, the effect of IBD on health-care systems will rise exponentially [25]. An estimated 2.5–3 million people in Europe are affected by IBD, with a direct healthcare cost of 4.6–5.6 billion Euros (EUR) per year [66]. Thus, annual healthcare costs were significantly higher for patients younger than 20 years compared with adults [68]. Expenses will most likely continue to rise, because individualized pediatric therapy, especially the new biologics, while becoming more effective, is also more expensive. The health care system and health insurance companies need current disease rates and projected patient numbers in order to optimize the care of IBD patients.

### Strengths and limitations

#### Strengths

The results are based on over 15 years of population-based registry data with excellent quality from all over Saxony. The registry has an estimated completeness level of 95.7% for children up to 15 years of age (nearly full census). Thus, the incidence trend of pediatric IBD in the federal state of Saxony has been reliably determined.

The challenge of differentiating between individual diagnoses is mastered more reliably with the length of treatment. The relatively long duration of the Saxon registry with the small number of final IBD-U cases (3.4%) confirms the diagnostic certainty of the study.

The problem of appropriate diagnosis (classification and evaluation of the patient in the correct diagnosis group) was given the highest attention. The quality of the registry data benefited both from the years of patient follow-up, and ultimately, also from the time interval between the survey period and publication–the query in 2018 was a significant step towards further validation. The first diagnosis made on the “Initial registration form” was not evaluated, rather the most frequent diagnosis in the most recent reports, which logically contains more information input.

The number of physician visits reported varied and so the level of information per IBD case was different. This is likely to have played only a minor role in the quality of the epidemiological evaluations presented here, including any considerations of exact diagnoses. All IBD-U cases were clarified as best as possible, and reciprocal change of diagnosis from CD to UC or vice versa was observed with approximately equal frequency in the registry. Pediatric gastroenterologists regularly reviewed diagnoses, modifying them as necessary. Sometimes individual patient data underwent a thorough review process by multiple pediatric gastroenterologists.

One of the strengths of our study is that all participating pediatric gastroenterologists regularly hold meetings, in order to promote registry participation. Procedures, diagnostics, and therapies were discussed, optimized, and unified at these meetings. Internal data validity controls within the database and an external re-assessment were carried out regularly.

#### Limitations

The low number of IBD-U diagnoses is very positive, as a result of generally mature diagnostic procedure. Nevertheless, all single IBD-U sub-results cannot represent any significant scientific weight due to the small number of cases und were only listed for documentation. In particular, the small numbers of cases resulted in wide credibility intervals for the projections. Therefore, the results should be interpreted as an indication of possible future developments rather than as a concrete prediction of the future.

The questionnaires were adapted and supplemented several times over the 15 years of operation, e.g., after the appearance of Porto Criteria and Montreal Classification 2005. Thus, some questionnaire sections were not included on earlier reporting forms. This could affect some future clinical but not our epidemiologic evaluations. Reports on a patient were usually compiled over many years, completed and validated periodically (also subsequently) to avoid a significant bias in the results.

## Conclusions

Pediatric IBD incidence rates are increasing significantly over time based on 15 years of complete and valid population-based, high quality data from the Saxon Pediatric IBD Registry. We were also able to determine expected trends in future patient numbers and estimate future treatment costs. Considering the model-based projection, the number of pediatric IBD patients in Germany can be expected to double in 2030 compared to annual average in the registry period 2000–2014. However, this is far from answering all research questions. Deeper exploration into the etiology of IBD would certainly contribute to a better understanding and more effective therapy of the disease.

## Supporting information

S1 Appendix Questionnaire initial registration form–original in German. (PDF)

S2 Appendix Questionnaire initial registration form–translation in English. (PDF)

S3 Appendix Questionnaire documentation form–original in German. (PDF)

S4 Appendix Questionnaire documentation form–translation in English. (PDF)

S5 Appendix Supplement of Table 1 –Age-standardized incidence rates (ASR). Annual rates 2000–2014.
(PDF)

S6 Appendix Supplement of Table 5 –Projected number of children and adolescents in Saxony to the years 2025 and 2030 in supplementary AG. (PDF)
