# Supplementary material for: Current and projected incidence trends of pediatric-onset inflammatory bowel disease in Germany based on the Saxon Pediatric IBD Registry 2000–2014 –a 15-year evaluation of trends
Source: PLoS One. 2022 Sep 9;17(9):e0274117. doi: 10.1371/journal.pone.0274117 (PMC9462751; doi:10.1371/journal.pone.0274117)
Supplement: S3 Appendix — (PDF) [file pone.0274117.s003.pdf]

Name, Vorname des Versicherten

geb. am

Kassen-Nr. | Versicherten-Nr. | Status

Betriebsstätten-Nr. | Arzt-Nr. | Datum

CHRONISCH ENTZÜNDLICHE DARMERKRANKUNGEN (CED) BEI KINDERN UND JUGENDLICHEN

Kinder-CED-Register Sachsen

DOKUMENTATIONSBOGEN

Vorstellungsdatum aktuell

Vorstellungsdatum vorheriges

Basisdaten

Vorstellungsart
- ○ ambulant
- ○ stationär

Untersuchungsanlass
- ○ geplant
- ○ akut

☐ **andere chronische Erkrankungen**

Diagnose **Wechsel** ☐
- ○ M. Crohn
- ○ Colitis ulcerosa
- ○ Colitis indeterminata

Größe [cm] ___ , ___

Gewicht [kg] ___ , ___

Knochenalter
___ , ___ Jahre
___ / ___ Erhebung: (Monat/Jahr)

Anamnese

Befinden
- ○ sehr gut
- ○ gut
- ○ mittel
- ○ schlecht
- ○ sehr schlecht

Appetit
- ○ gut
- ○ mäßig
- ○ schlecht

Alltagsaktivität
Einschränkung
- ○ keine
- ○ geringe
- ○ deutliche

Schulfehltage ___ **Tage**

Stuhlgang

Konsistenz
- ○ geformt
- ○ breiig
- ○ flüssig

Blut im Stuhl
- ○ nein
- ○ gelegentlich, wenig
- ○ meistens, wenig
- ○ viel

Anzahl
- ___ / tagsüber
- ___ / nachts

Bauchschmerzen
- ○ keine
- ○ leichte
- ○ mittelschwere
- ○ schwere
- ☐ nächtliche

Untersuchung

Abdominalbefund
- ○ **unauffällig**
- ☐ Druckschmerz
- ☐ Resistenz
- ☐ Abwehrspannung
- ☐ Stoma

Analbefund
- ○ unauffällig/reizlose Marisken
- ○ Rhagade/Fissur
- ○ inaktive Fistel
- ○ sezernierende Fistel/Abszess entzündliche Induration
- ☐ perianales Ekzem

Extraintestinale Symptome
- ○ **keine**
- ☐ Auge
- ☐ Fieber > 38,5°C > 3 Tage
- ☐ Gelenke: Schmerzen
- ☐ Haut
- ☐ Wirbelsäule
- ☐ Gelenke: Entzündung
- ☐ Leber/Gallenwege/Pankreas
- ☐ PSC
- ☐ andere: ___

Lippen- / Mundbeteiligung
○ nein ○ ja

Pubertät nach Tanner **PH** ___ (1-5) **B** ___ (1-5) **Hodenvolumen** ___ ml **Menarche** ○ nein ○ ja ___ (Jahr)

Labor

**Hämoglobin**	○ mmol/l ○ g/dl	CRP	○ mg/l ○ mg/dl	Lipase	○ µmol/l*s ○ U/l	Calprotectin	○ mg/kg ○ mg/l
**Hämatokrit**	○ ○ %	**BSG**	___ ___	MCV	○ fl ○ µm³	Laktoferrin	○ mg/kg ○ mg/l
Thrombozyten	○ Gpt/l ○ 1/µl	ALAT (GPT)	○ µmol/l*s ○ U/l	**Albumin i.S.**	○ g/l ○ g/dl		
**Leukozyten**	○ Gpt/l ○ 1/µl	Gamma-GT	○ µmol/l*s ○ U/l	Kreatinin i.S.	○ µmol/l ○ mg/dl		

Urinuntersuchung
- ○ n. durchgef.
- ○ unauff.
- ☐ Proteinurie
- ☐ Hämaturie

Apparative Diagnostik

- ○ **keine**
- ☐ Ileokoloskopie
- ☐ Rö-Sellink
- ☐ MRT Becken
- ☐ CT Bauch
- ☐ Ösophagogastroduodenoskopie
- ☐ Koloskopie
- ☐ MRT Darm
- ☐ Histologie
- ☐ Rektosigmoidoskopie
- ☐ Ultraschall
- ☐ andere: ___

Lokalisation

- ☐ unauffällig
- ☐ keine Änderungen
- ☐ keine neuen Informationen
- ☐ Ösophagus
- ☐ übriger Dünndarm
- ☐ Colon asc.
- ☐ Sigmoideum
- ☐ Magen
- ☐ term. Ileum
- ☐ Colon trans.
- ☐ Rektum
- ☐ Duodenum
- ☐ Zökum
- ☐ Colon desc.
- ☐ Pouch

Komplikationen / OP ○ **keine**
- ☐ Perianalfistel
- ☐ sonstige Fistel
- ☐ Perianalabszess
- ☐ sonstiger Abszess
- ☐ intraabdom. Absz.
- ☐ Stenose
- ☐ andere: ___
- ☐ OP: ___ Typ ___ Datum

Bisherige Therapie

○ **keine Therapie**

Oral / i.v. (in mg/d)
- SASP (Sulfasalazin) ___
- Budesonid ___
- 5-ASA (Mesalazin) ___
- ○ Azathioprin ○ 6-MP ___
- Kortikoide ○ **i.v.** ○ **p.o.** ___
- zwischenzeitig Steroidtherapie seit letzter Dokumentation ○ nein ○ ja

Ern. Therapie
- ☐ ausschließliche ○ **p.o.** ○ **per Sonde**
- ☐ nächtliche Sondierung
- ☐ kalorische Supplementierung
- ☐ Produkt: ___

Rektal
- ☐ 5-ASA (Mesalazin)
- ☐ Kortikoide
- ☐ Budesonid

andere
- ☐ Methotrexat
- ☐ Antibiotika
- ☐ Komplementärmedizin
- ☐ Eisen
- ☐ Cyclosporin A
- ☐ Probiotika
- ☐ Calcium/Vitamin D
- ☐ Vit. / Spuren.
- ☐ Infliximab
- ☐ Säureblocker
- ☐ andere: ___

Therapienebenwirkung ○ keine ○ ja: ___

Augenarzttermin seit letzter Vorstellung
- ○ nein
- ○ ja

Psychosoziale Therapie
- ○ nein
- ○ ja

nicht dokumentierte Vorstellungen seit letzter Dokumentation
___ (Anzahl der Vorstellungen)

Einverständniserklärung liegt vor ○ ja

Ärztliche Gesamtbeurteilung
- ○ Remission
- ○ leichte Aktivität
- ○ mittelschwere Aktivität
- ○ schwere Aktivität

Ambulanz-Nr.

Unterschrift:

(Version 01/08)
