# Supplementary material for: Current and projected incidence trends of pediatric-onset inflammatory bowel disease in Germany based on the Saxon Pediatric IBD Registry 2000–2014 –a 15-year evaluation of trends
Source: PLoS One. 2022 Sep 9;17(9):e0274117. doi: 10.1371/journal.pone.0274117 (PMC9462751; doi:10.1371/journal.pone.0274117)
Supplement: S4 Appendix — (PDF) [file pone.0274117.s004.pdf]

Automated, individual related data of the health insurance

INFLAMMATORY BOWEL DISEASES (IBD)
IN CHILDREN AND ADOLESCENTS
DOCUMENTATION FORM

Pediatric IBD Registry - Saxony -

Date of visit - actual - ☐☐☐☐☐☐

Date of previous visit ☐☐☐☐☐☐

Base data

Examination	Occasion	Diagnosis Change ☐
○ ambulatory	○ planned	○ Crohn's disease
○ stationary	○ acute	○ Ulcerative Colitis
☐ **other chronic diseases**		○ Unclassified IBD

Height [cm] ☐☐☐,☐

Weight [kg] ☐☐☐,☐

Bone Age: ☐,☐ Years ; ☐ / ☐ Inquiry date (month/year)

Clinical history

State of health	Appetite	Daily activity
○ very good	○ good	Restrictions
○ good	○ moderate	○ none
○ mediocre	○ bad	○ few
○ bad		○ significant
○ very bad		

Absence from school ☐☐☐ days

Stool

Consistency	Fecal Blood	Number
○ shaped	○ never	☐ /day
○ pulpy	○ occasionally, few	☐ /night
○ liquid	○ mostly, few	
	○ many	

Stomach Pains
- ○ none
- ○ slight
- ○ moderate
- ○ heavy
- ☐ overnight

Examination

Abdominal findings
- ○ **inconspicuous**
- ☐ pressure pain
- ☐ resistance
- ☐ muscular defense
- ☐ stoma

Anal findings
- ○ skin tags without irritation
- ○ rhagades/fissures
- ○ inactive fistula
- ○ secreting fistula/abscess inflamming Induration
- ☐ perianal eczema

Extraintestinal symptoms
- ○ **none**
- ☐ eyes
- ☐ skin
- ☐ pyrexia >38.5°C > 3 days
- ☐ spine column
- ☐ joints: pains
- ☐ joints: inflammation
- ☐ liver / bile tracts / pancreas
- ☐ PSC
- ☐ other: ____

Involvement a of lips / mouth ○ no ○ yes

Puberty (Tanner scale) PH ☐ (1-5) B ☐ (1-5) **Testicular volume** ☐ ml **Menarche** ○ no ○ yes ☐☐☐☐ (Year)

Laboratory results

hemoglobin ____ ○ mmol/l ○ g/dl	CRP ____ ○ mg/l ○ mg/dl	Lipase ____ ○ µmol/l*s ○ U/l	Calprotectin ____ ○ mg/kg ○ mg/l
hematocrit ____ ○ ○ %	ESR ____ | ____	MCV ____ ○ fl ○ µm³	Lactoferrin ____ ○ mg/kg ○ mg/l
thrombocytes ____ ○ Gpt/l ○ 1/µl	ALAT (GPT) ____ ○ µmol/l*s ○ U/l	Albumin i.S. ____ ○ g/l ○ g/dl	**Urinalysis**
leukocytes ____ ○ Gpt/l ○ 1/µl	Gamma-GT ____ ○ µmol/l*s ○ U/l	Creatinin i.S. ____ ○ µmol/l ○ mg/dl	○ not perf. ○ inconsp. ☐ Proteinuria ☐ Hematuria

Instrumental Diagnosis

- ○ **none**
- ☐ ileocolonoscopy
- ☐ X-ray-Sellink
- ☐ MRI pelvic region
- ☐ CT abdomen
- ☐ esophagogastroduodenoscopy
- ☐ coloscopy
- ☐ MRI bowel
- ☐ histology
- ☐ rectosigmoidoscopy
- ☐ sonography
- ☐ other: ____

Location

- ☐ inconspicuous
- ☐ no changes
- ☐ no new information
- ☐ esophagus
- ☐ stomach
- ☐ duodenum
- ☐ remaining small intestine
- ☐ terminal ileum
- ☐ cecum
- ☐ colon asc.
- ☐ colon trans.
- ☐ colon desc.
- ☐ sigmoideum
- ☐ rectum
- ☐ pouch

Complications / Surgery ○ none
- ☐ perianal fistula
- ☐ misc. fistula
- ☐ perianal abszess
- ☐ misc. abszess
- ☐ intraabdom. absz.
- ☐ Stenosis
- ☐ other: ____
- ☐ OP: ____ Date

Therapy

○ **no therapy**

Oral (i.a. mg/d)
- SASP (Sulfasalazin) ____
- Budesonid ____
- 5-ASA (Mesalazin) ____
- ○ Azathioprin ○ 6-MP ____
- Corticosteroids ○ **i.v.** ○ **p.o.** ____
- meantime steroid therapy since last documentation ○ no ○ yes

Nutrition therapy
- ☐ exclusive ○ **p.o.** ○ **p.tube.**
- ☐ nocturnal tube feeding
- ☐ caloric supplementation
- ☐ product: ____

Rectal
- ☐ 5-ASA (Mesalazin)
- ☐ Corticosteroids
- ☐ Budesonid

other
- ☐ Methotrexat
- ☐ Antibiotics
- ☐ Complementary medicine
- ☐ Cyclosporin A
- ☐ Probiotics
- ☐ Calcium/Vitamine D
- ☐ Iron
- ☐ Infliximab
- ☐ Antracid
- ☐ other: ____
- ☐ Vit./trace elem.

Adverse reaction to therapy ○ none ○ yes: ____

presentation at ophthalmologist since last visit ○ no ○ yes

Psychosocial Therapy ○ no ○ yes

Undocumented visits since last documented presentation ☐ (Number of visits)

Informed consent: available ○ yes

Medical overall assessment
- ○ Remission
- ○ Light activity
- ○ Moderate activity
- ○ Heavy activity

☐☐☐ *Outpatient department*

Signature: ____

(Version 01/08)
