# Supplementary material for: Current and projected incidence trends of pediatric-onset inflammatory bowel disease in Germany based on the Saxon Pediatric IBD Registry 2000–2014 –a 15-year evaluation of trends
Source: PLoS One. 2022 Sep 9;17(9):e0274117. doi: 10.1371/journal.pone.0274117 (PMC9462751; doi:10.1371/journal.pone.0274117)
Supplement: S5 Appendix — Annual rates 2000–2014. (PDF) [file pone.0274117.s005.pdf]

S5 Appendix. Age-standardized incidence rates (ASR) of inflammatory bowel disease (IBD), Crohn's disease (CD), ulcerative colitis (UC) and unclassified IBD (IBD-U) per 100,000 person-years (PY) for children and adolescents < 15 years of age at diagnosis in Saxony. Annual rates 2000-2014 and total rates per one year.

	population at risk	IBD			CD			UC			IBD-U		
		n	ASR	[95 % CI]	n	ASR	[95 % CI]	n	ASR	[95 % CI]	n	ASR	[95 % CI]
**year**													
2000	550,835	30	**4.6**	[2.8 - 6.3]	21	**3.3**	[1.8 - 4.8]	9	**1.3**	[0.4 - 2.1]	0	**0.0**	-
2001	522,225	32	**4.7**	[3.0 - 6.3]	15	**2.2**	[1.1 - 3.3]	15	**2.2**	[1.1 - 3.3]	<5	**0.3**	[0.0 - 0.7]
2002	494,070	42	**7.7**	[5.3 - 10.1]	27	**5.0**	[3.1 - 7.0]	14	**2.5**	[1.2 - 3.8]	<5	**0.2**	[0.0 - 0.5]
2003	470,594	38	**7.5**	[5.1 - 9.9]	25	**5.0**	[3.0 - 6.9]	13	**2.6**	[1.2 - 4.0]	0	**0.0**	-
2004	451,952	43	**9.8**	[6.9 - 12.8]	28	**6.4**	[4.0 - 8.7]	15	**3.5**	[1.7 - 5.2]	0	**0.0**	-
2005	436,305	25	**6.3**	[3.8 - 8.8]	16	**4.0**	[2.0 - 6.0]	7	**1.8**	[0.5 - 3.1]	<5	**0.5**	[0.0 - 1.3]
2006	437,421	31	**8.0**	[5.2 - 10.8]	19	**5.0**	[2.7 - 7.3]	11	**2.8**	[1.1 - 4.4]	<5	**0.2**	[0.0 - 0.6]
2007	444,508	22	**5.8**	[3.3 - 8.2]	12	**3.2**	[1.4 - 5.0]	10	**2.6**	[1.0 - 4.2]	0	**0.0**	-
2008	454,198	35	**8.3**	[5.5 - 11.0]	23	**5.4**	[3.2 - 7.7]	11	**2.6**	[1.0 - 4.1]	<5	**0.3**	[0.0 - 0.8]
2009	464,584	46	**10.5**	[7.5 - 13.6]	26	**6.1**	[3.7 - 8.4]	17	**3.8**	[2.0 - 5.6]	<5	**0.7**	[0.0 - 1.5]
2010	476,168	33	**7.5**	[4.9 - 10.0]	23	**5.1**	[3.0 - 7.2]	10	**2.3**	[0.9 - 3.8]	0	**0.0**	-
2011	480,220	33	**7.5**	[4.9 - 10.0]	20	**4.6**	[2.6 - 6.6]	13	**2.9**	[1.3 - 4.5]	0	**0.0**	-
2012	488,364	45	**9.8**	[6.9 - 12.7]	34	**7.5**	[5.0 -10.0]	8	**1.7**	[0.5 - 2.9]	<5	**0.6**	[0.0 - 1.3]
2013	496,028	38	**8.2**	[5.6 - 10.8]	25	**5.4**	[3.3 - 7.5]	12	**2.6**	[1.1 - 4.1]	<5	**0.2**	[0.0 - 0.7]
2014	504,802	39	**8.2**	[5.6 - 10.8]	24	**5.1**	[3.0 - 7.1]	11	**2.4**	[1.0 - 3.7]	<5	**0.8**	[0.0 - 1.6]
**Total**	**7,172,274**	**532**	**7.5**	**[6.7 - 8.1]**	**338**	**4.8**	**[4.3 - 5.3]**	**176**	**2.5**	**[2.1 - 2.9]**	**18**	**0.3**	**[0.1 - 0.4]**

Legend: source of population data [31].
