# Supplementary material for: Current and projected incidence trends of pediatric-onset inflammatory bowel disease in Germany based on the Saxon Pediatric IBD Registry 2000–2014 –a 15-year evaluation of trends
Source: PLoS One. 2022 Sep 9;17(9):e0274117. doi: 10.1371/journal.pone.0274117 (PMC9462751; doi:10.1371/journal.pone.0274117)
Supplement: S6 Appendix — (PDF) [file pone.0274117.s006.pdf]

S6 Appendix. Projected number of children and adolescents <15 years of age at diagnosis with inflammatory bowel disease (IBD), Crohn's disease (CD) und ulcerative colitis (UC) per 100,000 person-years (PY) in Saxony to the years 2025 and 2030 (and by age and sex).

Cohort	Population at risk	Year	IBD		CD		UC	
			n	[95 % CrI]	n	[95 % CrI]	n	[95 % CrI]
**Supplementary AG**								
M-AG 0-4	90,000	2025	**3.3**	[3.0 – 3.6]	**2.4**	[2.1 – 2.7]	**0.6**	[0.5 – 0.7]
	84,000	2030	**3.6**	[3.2 – 4.0]	**2.7**	[2.3 – 3.1]	**0.6**	[0.5 – 0.8]
M-AG 5-9	96,000	2025	**10.2**	[9.3 – 11.0]	**7.4**	[6.7 – 8.2]	**2.1**	[1.8 – 2.4]
	91,000	2030	**11.4**	[10.2 – 12.6]	**8.5**	[7.4 – 9.6]	**2.2**	[1.8 – 2.6]
F-AG 0-4	85,000	2025	**2.3**	[2.1 – 2.5]	**1.5**	[1.3 – 1.7]	**0.6**	[0.5 – 0.7]
	80,000	2030	**2.5**	[2.2 – 2.8]	**1.7**	[1.4 – 1.9]	**0.6**	[0.5 – 0.7]
F-AG 5-9	92,000	2025	**7.2**	[6.6 – 7.9]	**4.7**	[4.2 – 5.2]	**2.0**	[1.7 – 2.3]
	87,000	2030	**8.1**	[7.2 – 8.9]	**5.4**	[4.6 – 6.1]	**2.0**	[1.7 – 2.4
AG 0-4	175,000	2025	**5.5**	[5.2 – 5.9]	**3.9**	[3.6 – 4.2]	**1.2**	[1.1 – 1.4]
	164,000	2030	**6.1**	[5.6 – 6.5]	**4.4**	[3.9 – 4.8]	**1.2**	[1.1 – 1.4]
AG 5-9	188,000	2025	**17.4**	[16.4 – 18.5]	**12.1**	[11.2 – 13.1]	**4.1**	[3.7 – 4.5]
	178,000	2030	**19.4**	[18.0 – 20.9]	**13.9**	[12.5 – 15.2]	**4.2**	[3.6 – 4.8]

Legend: M = male, F = female; source of population data [32].
